# Clinical Evaluation of In-House-Produced 3D-Printed Nasopharyngeal Swabs for COVID-19 Testing

**DOI:** 10.3390/v13091752

**Published:** 2021-09-02

**Authors:** Simon Grandjean Lapierre, Stéphane Bedwani, François DeBlois, Audray Fortin, Natalia Zamorano Cuervo, Karim Zerouali, Elise Caron, Philippe Morency-Potvin, Simon Gagnon, Nakome Nguissan, Pascale Arlotto, Isabelle Hardy, Catherine-Audrey Boutin, Cécile Tremblay, François Coutlée, Jacques de Guise, Nathalie Grandvaux

**Affiliations:** 1 Centre de Recherche du Centre Hospitalier de l’Université de Montréal (CRCHUM), 900 rue Saint-Denis, Montréal, QC H2X 0A9, Canada; simon.grandjean.lapierre@umontreal.ca (S.G.L.); stephane.bedwani.chum@ssss.gouv.qc.ca (S.B.); francois.deblois.chum@ssss.gouv.qc.ca (F.D.); audray.fortin.chum@ssss.gouv.qc.ca (A.F.); natalia.zamorano@umontreal.ca (N.Z.C.); karim.zerouali.chum@ssss.gouv.qc.ca (K.Z.); elise.caron@crchum.qc.ca (E.C.); philippe.morency-potvin.med@ssss.gouv.qc.ca (P.M.-P.); simon.gagnon.chum@ssss.gouv.qc.ca (S.G.); nakome.nguissan.chum@ssss.gouv.qc.ca (N.N.); pascale.arlotto.chum@ssss.gouv.qc.ca (P.A.); isabelle.hardy.chum@ssss.gouv.qc.ca (I.H.); c.tremblay@umontreal.ca (C.T.); francois.coutlee.med@ssss.gouv.qc.ca (F.C.); jacques.deguise@etsmtl.ca (J.d.G.); 2 Department of Microbiology, Infectious Diseases and Immunology, Université de Montréal, Montréal, QC H3C 3J7, Canada; catherine-audre.boutin@mail.mcgill.ca; 3 Department of System Engineering, École de Technologie Supérieure, Université du Québec, Montréal, QC H3C 1K3, Canada; 4 Department of Biochemistry and Molecular Medicine, Faculty of Medicine, Université de Montréal, Montréal, QC H3C 3J7, Canada

**Keywords:** SARS-CoV-2, COVID-19, 3D-printed nasopharyngeal swabs, diagnosis, PCR

## Abstract

3D-printed alternatives to standard flocked swabs were rapidly developed to provide a response to the unprecedented and sudden need for an exponentially growing amount of diagnostic tools to fight the COVID-19 pandemic. In light of the anticipated shortage, a hospital-based 3D-printing platform was implemented in our institution for the production of swabs for nasopharyngeal and oropharyngeal sampling based on the freely available, open-source design provided to the community by University of South Florida’s Health Radiology and Northwell Health System teams as a replacement for locally used commercial swabs. Validation of our 3D-printed swabs was performed with a head-to-head diagnostic accuracy study of the 3D-printed “Northwell model” with the cobas PCR Media^®^ swab sample kit. We observed an excellent concordance (total agreement 96.8%, Kappa 0.936) in results obtained with the 3D-printed and flocked swabs, indicating that the in-house 3D-printed swab could be used reliably in the context of a shortage of flocked swabs. To our knowledge, this is the first study to report on autonomous hospital-based production and clinical validation of 3D-printed swabs.

## 1. Introduction

On 11 March 2020, the World Health Organization (WHO) declared the coronavirus 19 disease (COVID-19) outbreak caused by the highly transmissible severe acute respiratory syndrome coronavirus 2 (SARS-CoV-2) a global pandemic [1,2,3,4]. This triggered an unprecedented and sudden need for an exponentially growing amount of personal protection equipment and diagnostic tools. Real-time polymerase chain reaction (PCR)-based detection of SARS-CoV-2 nucleic acids rapidly became the gold standard to diagnose patients infected with SARS-CoV-2 [5]. As typically performed for the detection of respiratory viruses, an accurate COVID-19 PCR assay relies on the collection of samples from the upper respiratory tract, including nasopharyngeal (NP) and oral mucosal surfaces [6]. Flocked swabs feature perpendicular fibers that optimize specimen collection and elution in transport media, and are hence considered optimal for sampling the respiratory tract mucosal surfaces. As testing rapidly became critical for the development of a COVID-19 response strategy, the world encountered a shortage of PCR reagents and sampling swabs, resulting in testing backlogs, delayed diagnoses, compromised contact tracing and quarantine of patients, and potentially increased disease transmission.

The versatility of 3D printing, as well as the possibility of rapidly developing prototypes, have enabled rapid mobilization of this technology to provide a response to the interruption of supply chains [7,8]. The 3D-printed alternatives to standard flocked swabs were rapidly developed. Early in the pandemic, an open-source design for 3D-printed swabs was generously made available to the community by the teams from the University of South Florida’s (USF) Health Radiology and Northwell Health System (NHS) [9,10]. The design and workflow for hospital-based printing was subsequently published [8]. Local manufacturing based on 3D printing is among the strategies that can help alleviate supply chain shortages.

In light of the anticipated shortage of sampling swabs in our hospital and in the province of Quebec, Canada, we sought to locally manufacture and evaluate sterile 3D-printed swabs based on the freely available open-source designs [8]. Here, we report on the fabrication process and clinical evaluation of 3D-printed swabs against the cobas PCR Media^Ⓡ^ swab sample kit in a prospective cohort of symptomatic healthcare workers. To our knowledge, this is the first study to report on the evaluation of autonomous hospital-based production of 3D-printed swabs. Our clinical evaluation showed that the locally printed swabs were a reliable alternative to commercial swabs. These results support the initial assumption made by the USF and NHS groups that 3D-printed swabs produced in hospitals can be a rapid local response to meet demand in the event of a disruption in swab supply chains due to the pandemic.

## 2. Materials and Methods

### 2.1. 3D Printing

The 3D prototypes of swabs were designed and shared by teams from the Division of 3D Clinical Applications at USF and NHS [8]. All final printed models successfully passed a complete set of mechanical tests [11]. In our hospital-based production, we used the model referred to as the “Northwell model”, to which a breaking point located 80 mm from the tip was added to the original design.

Swabs were printed by stereolithography (SLA) using Formlabs Form 3 and 3B printers with Surgical Guide resin (Formlabs, Mississauga, ON, Canada, Cat#RS-F2-SGAM-01) that was biocompatible and sterilizable. The Preform software (Formlabs) was used to create an array of 256 swab models to be converted into printer instructions. The thickness of each printed layer was set to 0.1 mm. Printed swabs were cleaned in 99% isopropyl alcohol (Sigma-Aldrich, Saint Louis, MO, USA, Cat#PX1835-3) for 20 min using the Form Wash (Formlabs) station and allowed to air dry for at least 30 min before being post-cured at 70 °C for 30 min in a Form Cure (Formlabs) station. Visual inspection was performed to verify that printed swabs were free of defects, otherwise they were discarded.

### 2.2. Sterilization

The 3D-printed swabs were immediately individually packed in autoclavable flat pouches (4 in × 10.5 in, Stevens Company, Anjou, QC, Canada, #164-S5) previously identified (lot number and date of production) with autoclave-resistant laser labels (GA International, Laval, QC, Canada, #AKA-13). Pouches were sealed with a vacuum rotosealer machine (Wipak Medical, Welshpool, United Kingdom, #RS120) and inspected visually. Sterilization was performed by autoclaving using a pre-vacuum steam cycle set at 132 °C (270 °F) for 4 min, followed by a 30 min drying period.

To verify the efficacy of the sterilization, the head of two swabs per production lot were inoculated with 10 μL of the biological indicator *Geobacillus stearothermophilus* spore suspension (1.7 × 10^7^ CFU/0.1 mL; Steris Corporation, Mentor, OH, USA, #NA-091) under sterile conditions [12]. Inoculated swabs were held horizontally for 30 min and further allowed to dry vertically for 24 h under sterile conditions. Next, dried swabs were sealed individually in sterilization pouches. One of the inoculated swabs was subjected to sterilization as described above, together with all swabs from the same lot. The other inoculated swab was kept in the pouch and was not sterilized. The two inoculated swabs were broken at the breakpoint and inserted into a bacteriology tube (Sarstedt, Montreal, QC, Canada, #62515006) containing 5 mL of tryptic soy broth culture media (Sigma-Aldrich, #1463170010). Bacteria growth was monitored by incubation at 55 °C with orbital agitation for 7 days. A culture tube containing culture media was used as negative control. OD600 nm was measured on day 7 to assess bacterial growth.

### 2.3. Quality Control

Swabs showing excessive warping post-sterilization were discarded after visual inspection. Basic mechanical testing was performed using a guide formed by 3 semicircular canals with a minimum radius of 15, 25, and 35 mm, respectively, which allowed testing the flexibility of swabs. The test was successful if the head and neck remained intact after passing through each canal. The final test consisted of breaking the swab in half with one hand at its breaking point.

### 2.4. 3D Swab Clinical Evaluation and Study Participant Recruitment

This study was performed in our institution’s COVID-19 rapid screening clinic and included symptomatic healthcare workers self-presenting for COVID-19 diagnostic testing. All participants provided written informed consent. During an initial recruitment phase, participants were tested simultaneously with both the cobas PCR Media^®^ swab sample kit (Roche Diagnostics, Florham Park, NJ, USA) and the 3D-printed swabs. As for any COVID-19 test, all results were transmitted to the institution’s occupational health and safety office and public health authorities. In a subsequent recruitment phase, to increase the number of positive samples within the study, patients having previously tested positive on routine testing were contacted by clinical research personnel and offered to participate in the study. For those participants, both sampling techniques were repeated simultaneously.

### 2.5. Oro-Nasopharyngeal Swab Collection and SARS-CoV-2 PCR Testing

A sequential oropharyngeal and nasopharyngeal sampling with each single swab was performed. The swab from the cobas PCR Media^®^ swab sample kit and the 3D-printed swab were used in the same nostril in a randomized order. The swabs were transferred separately to tubes containing the cobas PCR Media^®^ transport medium before proceeding with the PCR analysis. All samples were tested on the FDA emergency use authorization (EUA)-approved and locally validated cobas 8800 automated RT-PCR system (Roche Diagnostics), which simultaneously tests the *ORF1 a*/*b* and *E* gene viral molecular targets together with an internal control [13].

### 2.6. Statistical Analysis

The Lilliefors statistical test adapted from the Kolmogorov–Smirnov nonparametric test was used to assess data distribution normality. The Wilcoxon matched-pairs test for non-normal distributions was used to evaluate the difference between means of RT-PCR cycle thresholds (Ct) obtained following both sampling methods. Concordance analysis between both assays using overall, positive, and negative agreement percentages was performed with calculation of the Cohen’s Kappa values. By definition, Kappa values above 0.75 indicate excellent agreement, values between 0.40 and 0.75 indicate fair to good agreement, and values below 0.40 represent poor agreement beyond chance [14]. Results obtained with the cobas PCR Media^®^ swab sample kit were considered as the reference for positive and negative agreement calculation purposes.

## 3. Results

### 3.1. 3D-Printed Swab Model

**Figure 1:**
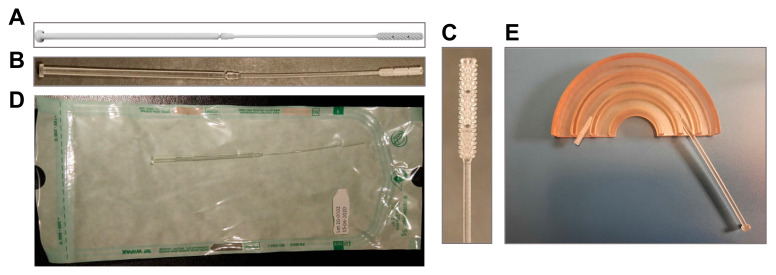
In-house 3D-printed swab model. Design of the Northwell 3D swab model with the addition of a breakout point (**A**) used to 3D-print swab in our hospital (**B**,**C**). Swabs were individually packed in autoclavable and vacuum-sealed pouches (**D**) for sterilization. (**E**) Flexibility was mechanically tested using semicircular canals (radius of 15, 25, and 35 mm).

**Figure 2:**
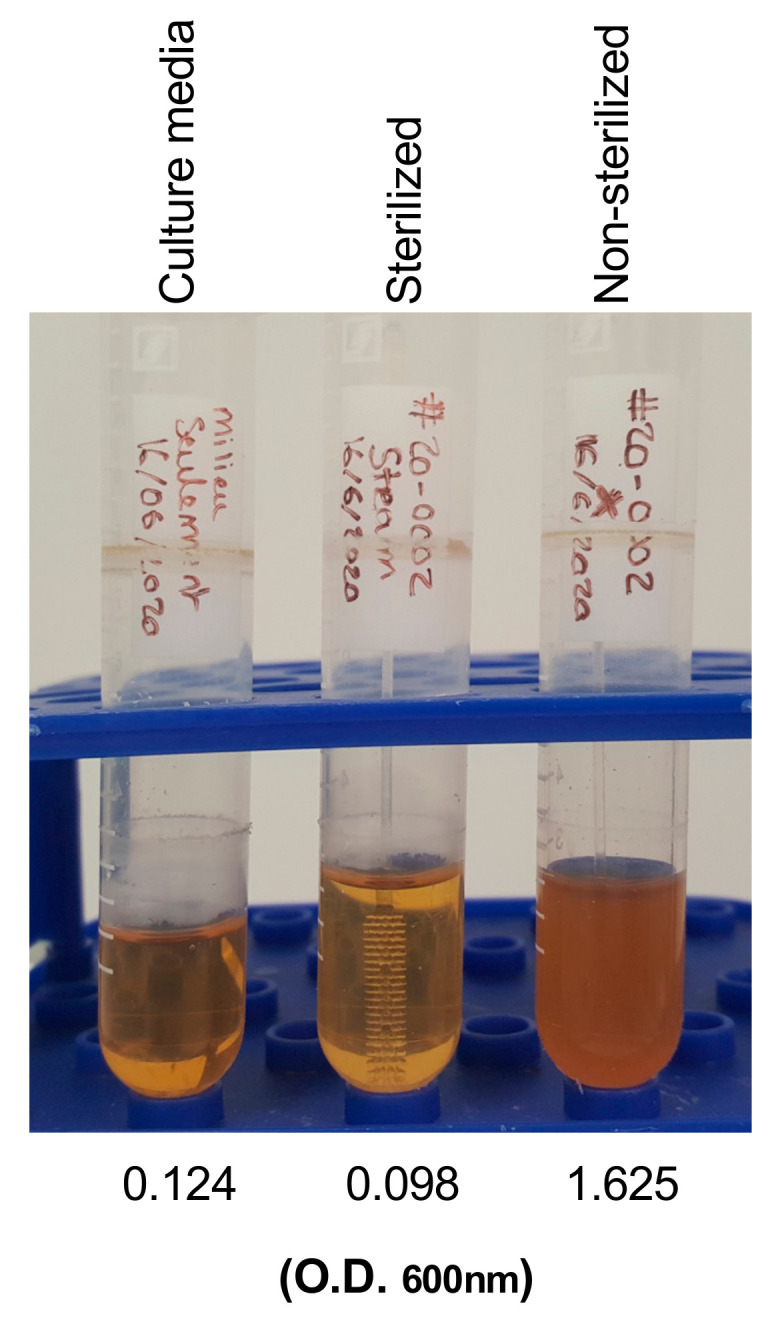
Validation of 3D-printed swab sterilization. Swab heads inoculated with *G. stearothermophilus* spore suspension before sterilization cultured in soy broth culture media. Bacteria growth was assessed by measuring the optical density (O.D.) at 600 nm. Culture media alone was used as negative control.

In a pilot print to begin implementing the hospital production of 3D-printed swabs, a biocompatible and sterilizable Surgical Guide resin was used to print two models, referred to as “USF” and “Northwell” [8], from the designs shared by the 3D Clinical Applications Division of USF and NHS. Based on the flexibility and the smaller head size, the “Northwell model” was selected by the clinical diagnostic team to use in the clinical tests. The cattail design of the “Northwell” model is composed of a head (18.0 mm long and 3.3 mm in diameter), a flexible neck (56.0 mm; 1.2 mm), and a handle (77.0 mm; 2.6 mm). Based on this pilot evaluation, a breaking point (1.0 mm; 1.4 mm) located at 80 mm from the tip to the original design was added to facilitate the release of the swab head in the transport tube containing the transport medium (Figure 1A–C). Three separate lots of 256 swabs were printed to ensure reproducibility of the production process and of the subsequent clinical evaluation. Swabs were packed individually in sterilization pouches before autoclaving (Figure 1D). Efficiency of the sterilization was verified for each lot through inhibition of *Geobacillus stearothermophilus* spore suspension inoculated on the head of one swab per lot (Figure 2). Two swabs from each printed lot were subjected to a quality-control check, including testing of the flexibility (Figure 1E) and breaking of the swabs at the breaking point. Overall, we observed a rate of 3.6% of swabs that had to be discarded based on visual inspection post-sterilization.

### 3.2. Clinical Testing

**Figure 3:**
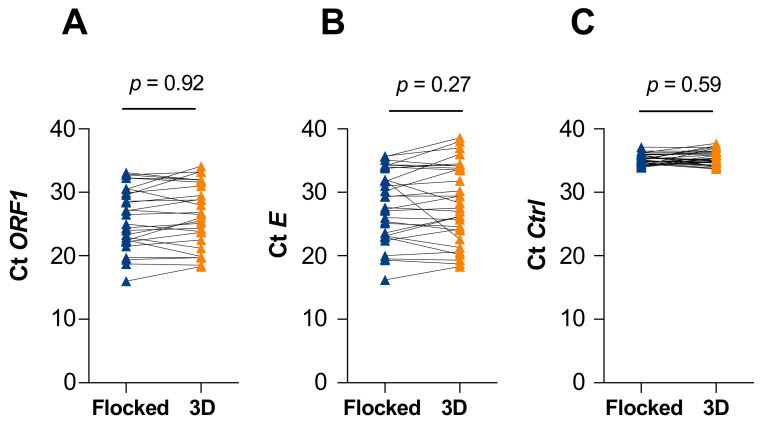
Cycle threshold (Ct) values of reverse-transcriptase polymerase chain reaction (RT-PCR) for the ORF1 and E genes. Participants were swabbed in the same nostril with a flocked and a 3D-printed swab, successively. RT-PCR was performed to measure the Ct values of *ORF1* (**A**) and *E* (**B**) viral genes for each swab. An internal control (Ctrl) was also included (**C**). Statistical analyses are detailed in Table 1.

A total of 63 participants were enrolled in the study. Thirty-two participants tested negative and 31 tested positive with the cobas 8800 SARS-CoV-2 PCR. Swabs from the three distinct printed lots were used for sample collection (batch 1, n = 21, 11 positives, 10 negatives; batch 2, n = 21, 11 positives; 10 negatives; batch 3, n = 21, 9 positives, 12 negatives). The Lilliefors statistical test and Kolmogorov–Smirnov nonparametric test showed that cycle threshold (Ct) distributions for both swabs were not standard (*p* = 0.12). PCR results for the *E* gene (*p* = 0.27), *ORF1* gene (*p* = 0.92), and internal control (*p* = 0.59) did not show significant Ct differences between flocked swabs and 3D-printed swabs (Figure 3 and Table 1).

A full agreement table between the flocked and the 3D-printed swabs is presented in Table 2 (A). Overall, positive and negative agreements were respectively 96.8% (61/63), 96.9% (31/32), and 96.8% (30/31), with a Kappa value of 0.936 (Table 2 (B)). In two cases, results obtained with the two swabs were discordant. In one case, the flocked swab sample was positive, with a Ct of 37.3 for the *E* gene only. For this same participant, the sample obtained with the 3D-printed swab led to no amplification for both genes. In the second discordant case, the flocked swab sample was negative for both genes, while the 3D-printed swab result was positive for both *ORF1* (Ct = 33.2) and *E* (Ct = 38.1) targets. However, in both discordant cases, the detected Ct were high, and several other samples included in this study also had Ct in the high range without showing discordant results.

## 4. Discussion

Here, we described the implementation of a hospital-based 3D-printing platform for the production of swabs for nasopharyngeal and oropharyngeal sampling for COVID-19 diagnosis. Such swabs are classified as Class I medical devices under the Canadian regulatory framework of Health Canada. To validate our 3D-printed swabs, we performed a head-to-head diagnostic accuracy study of the 3D-printed “Northwell model” swab [8] with the cobas PCR Media^®^ swab sample kit. We observed a high concordance (total agreement 96.8%, Kappa 0.936) in results obtained with the 3D-printed and flocked swabs, indicating that the in-house 3D-printed “Northwell model” swab could be used reliably in the context of a shortage of flocked swabs. The observed rate of positive and negative agreement was driven by results’ discrepancies suggesting increased sensitivity for both the flocked (flocked+/3D-) and the 3D (flocked-/3D+) swabs. Previous clinical trials performed by the USF and NHS teams reached similar conclusions by comparing the 3D-printed “USF model” swab with standard flocked swabs using alternative transport medium, including the WHO-approved viral transport media, media produced in-house according to the procedure described by the Centers for Disease Control and Prevention (CDC) or commercially available universal transport media [10]. At the beginning of our clinical trial, our hospital was not facing a shortage of supply, of swabs for the diagnosis of COVID-19, or planning to be out of stock in the medium term. Therefore, we were able to use the transport medium provided in the cobas PCR Media^®^ swab sample kit with the 3D-printed swab to ensure consistency in the clinical evaluation. The RT-PCR was performed through a trial on a single local cobas 8800 automated RT-PCR system authorized by Health Canada under an interim order for use related to COVID-19 diagnosis after complete published validation of the assay [13,15]. Given the complexity and discomfort associated with repeated simultaneous nasopharyngeal testing and the necessity to validate the use of 3D-printed swabs in a controlled head-to-head approach, we did not include other transport media in our validation study. We did not observe significant differences in Ct values between swabs for the *ORF1* and *E* SARS-CoV-2 genes in the Roche cobas assay. These observations were in agreement with the data from the clinical trial performed by the USF and Northwell system teams using the 3D-printed “USF model” swab compared to the Roche cobas sampling kit (97.03% agreement, Kappa 0.863) [10]. In a study comparing swabs from four distinct 3D-printing manufacturers to the Copan swab (501CS01) using an RT-PCR run on a Abbott m2000 RealTime system, a high degree of concordance with Kappa values between (0.85–0.89) was observed [16]. An additional prospective clinical validation compared 3D-printed swabs from two manufacturers with the Universal Viral Transport Kit by Becton, Dickinson & Company using a liaison MDX RT-PCR machine (DiaSorin Molecular, LLC) and the Simplexa COVID-19 Direct Kit (DiaSorin Molecular, LLC). Again, an excellent concordance between sampling procedures was observed [17].

To our knowledge, this is the first study reporting on autonomous hospital-based production and evaluation of 3D-printed swabs. The implementation of our hospital-based platform was facilitated by the existing “Health-related 3D printing centre” and access to an institutional sterilization service that allowed us to perform the steam sterilization that was chosen as a rapid, nontoxic and inexpensive technique that is microbicidal and sporicidal, and was previously shown to be compatible with the Surgical Guide resin [18]. The total cost per swab was about USD 0.56, which included USD 0.26 for consumables and USD 0.29 for staff wages. Further optimization has yet to be realized to increase the production volume and lower the production costs.

In conclusion, our study adds to the few clinical validation studies that demonstrated safety and accuracy of 3D-printed swabs. Our study is unique in that it tested a fully integrated hospital-based production of 3D-printed swabs as initially suggested by the USF and Northwell Health system groups. Our clinical trial demonstrated that our local 3D-printed swab production line offers a reliable local alternative to commercial swabs, and therefore confirmed that it is a viable local response to provide replacements in the event of pandemic supply-chain disruption. The option of in-house production of 3D-printed swabs remains particularly relevant, as the need for testing capacity continues to increase across the world, as many countries are experiencing new waves of infection with the emergence of variants of SARS-CoV-2 [19,20]. Our experience in the rapid implementation of this production line could serve as an example for other institutions around the world in the fight against COVID-19.

## Figures and Tables

**Table 1:** Mean Ct values of samples collected from individuals tested with a flocked or a 3D-printed swab.

	Flocked Mean Ct	3D Mean Ct	Delta Ct	*p*-Value ^1^
***ORF1***	26.06	26.51	0.44	0.92
***E***	28.03	28.30	0.26	0.27
***Ctrl***	35.16	35.35	0.19	0.59

^1^ Difference between means was evaluated with the Wilcoxon matched-pairs test.

**Table 2:** Tables of agreement between flocked and 3D-printed swabs showing positive and negative concordance percentages with Kappa values (**A**) and total number of positive (+), negative (-), and inconclusive (IC) PCR results (**B**).

A					
		3D	3D	3D	Total
		+	-	not conclusive	
**Flocked**	+	30	1	0	31
**Flocked**	-	1	31	0	32
**Flocked**	not conclusive	0	0	0	0
**Total**		31	32	0	63
**B**					
**Concordance**		**%**		**95% CI ^1^**	
**Positive**		96.8		82.4–99.9	
**Negative**		96.9		82.9–99.9	
**Total**		96.8		88.5–99.8	
**Kappa value**		0.936		0.738–0.994	

^1^ CI: confidence interval.

## Data Availability

Data is contained within the article.
